# Quantitative Analysis of Mixed Minerals with Finite Phase Using Thermal Infrared Hyperspectral Technology

**DOI:** 10.3390/ma16072743

**Published:** 2023-03-29

**Authors:** Meixiang Qi, Liqin Cao, Yunliang Zhao, Feifei Jia, Shaoxian Song, Xinfang He, Xiao Yan, Lixue Huang, Zize Yin

**Affiliations:** 1 Hubei Key Laboratory of Mineral Resources Processing and Environment, Wuhan University of Technology, 122 Luoshi Road, Wuhan 430070, China; 2 School of Resources and Environmental Engineering, Wuhan University of Technology, 122 Luoshi Road, Wuhan 430070, China; 3 Research Center of Graphic Communication, Printing and Packaging, Wuhan University, Wuhan 430079, China; 4 Xinjiang Lop Nur Potash Co., Ltd., 470 Tuanjie Road, Ruoqiang County, Bayingolin Mongolian Autonomous Prefecture 841800, China

**Keywords:** thermal infrared hyperspectral, potassium, quantitative analysis, finite mineral facies

## Abstract

It is crucial but challenging to detect intermediate or end products promptly. Traditional chemical detection methods are time-consuming and cannot detect mineral phase content. Thermal infrared hyperspectral (TIH) technology is an effective means of real-time imaging and can precisely capture the emissivity characteristics of objects. This study introduces TIH to estimate the content of potassium salts, with a model based on Competitive Adaptive Reweighted Sampling (CARS) and Partial Least Squares Regression (PLSR). The model takes the emissivity spectrum of potassium salt into account and accurately predicts the content of Mixing Potassium (MP), a mineral mixture produced in Lop Nur, Xinjiang. The main mineral content in MP was measured by Mineral Liberation Analyzer (MLA), mainly including picromerite, potassium chloride, magnesium sulfate, and less sodium chloride. 129 configured MP samples were divided into calibration (97 samples) and prediction (32 samples) sets. The CARS-PLSR method achieved good prediction results for MP mineral content (picromerite: correlation coefficient of correction set ($R_{p}^{2}$) = 0.943, predicted root mean square error (RMSEP) = 2.72%, relative predictive deviation (RPD) = 4.24; potassium chloride: $R_{p}^{2}$ = 0.948, RMSEP = 2.86%, RPD = 4.42). Experimental results convey that TIH technology can effectively identify the emissivity characteristics of MP minerals, facilitating quantitative detection of MP mineral content.

## 1. Introduction

During the mineral production process, the composition of intermediate and terminal products can change within a certain range due to factors such as alterations in the original ore’s nature, production operations, or other variables. In this type of product, although the mineral content varies within a certain range, the types of minerals are basically unchanged and can be referred to as “finite phase minerals”. Not adjusting production parameters in a timely manner based on actual production conditions can cause production instability, leading to a waste of resources and increased economic costs. As a result, it is essential to conduct intermittent sampling and tests to gather production information that can aid in the production process [1]. The chemical volumetric method is the primary analytical technique in production. In this method, minerals are dissolved, and specific chemical reagents are used to identify relevant elements. Although this method can meet production requirements to some extent, the low detection speed and incapable of detecting mineral phases cannot be neglected. Other mineral quantitative detection methods [2,3], such as Atomic Absorption Spectrometry (ASS) [4] and X-ray Fluorescence spectrometry (XRF) [5], can also quantitatively analyze and calculate the mineral element or ion content. However, these methods are still time-consuming and currently in the laboratory application stage.

Prompt-gamma neutron activation analysis [6] and infrared spectroscopy [7] are widely used and well-developed techniques for the rapid detection of quantitative analysis. They can be classified into two categories based on the presence of radioactivity. The first is nuclear radiation’s prompt-gamma neutron activation analysis technology, which excites atoms in the substance under test to release gamma rays for rapid detection of various elements. Although this detection technique can be widely used for the rapid detection of elemental and ion content in various minerals, its detection ability for mixed minerals containing the same ion is limited. As a result, the information provided by these techniques may be insufficient to accurately guide actual production. The second category is non-nuclear radiation’s infrared spectroscopy, which uses the spectral characteristics of reflected light from substances, combined with a quantitative predictive model [8,9], to achieve rapid detection of many minerals.

The infrared spectrum is divided into multiple intervals, and each interval has a specific response mechanism to the mineral’s ion and group spectrum [10], affecting the recognizing ability of minerals. The electronic processes of some metal ions (Fe^2+^, Fe^3+^, Cr^3+^, Mn^3+^, rare earth, etc.) are mainly detected in the visible to near-infrared bands [11]. The short-wave infrared bands primarily detect the double and harmonic frequencies of molecular vibrations of aqueous hydroxyl minerals [12,13] such as clay minerals, carbonate, and a few hydrated sulfates. The thermal infrared bands [14] mainly detect the fundamental frequency of molecular vibration, which has a good recognition effect on anhydrous and hydroxy-free minerals, such as silicate, carbonate, and sulfate. Compared with short-wave infrared, Thermal Infrared Technology (TIH) [15,16] shows a stronger capability for the detection of minerals containing atomic groups such as SinO_k_, SO_4_, CO_3_, and PO_4_. TIH [17,18] can detect the fundamental frequency vibration of atomic groups and their minor changes, making it easy to distinguish and identify silicates, sulfates, carbonates, phosphates, oxides, hydroxides, and other minerals. For instance, sulfate [19] has a characteristic absorption peak at 8.5 μm, and halides have a wide and slow double absorption peak between 5–10 μm. Potassium salt contains a large number of sulfate and chloride ions, which have noticeable characteristics in the thermal infrared bands. Therefore, the application of TIH technology in the rapid detection of potassium salt has theoretical feasibility.

TIH technology is a promising production monitoring technology due to its fast, safe, accurate, and stable characteristics. Emissivity [20] is the inherent property of substances, which can be obtained by TIH technology. High-precision emissivity data can be adopted to conduct qualitative and quantitative research on various minerals. Many studies have been carried out on the quantitative analysis of mineral content based on TIH techniques. An automatic recognition system [21] was proposed for common minerals such as garnet, olivine, and quartz in ground spectra based on long-wave infrared (7.7–11.8 μm) technology, achieving a high identification accuracy of 84.91% using cluster analysis. The Fe concentration was estimated based on long-wave infrared technology and data fusion method, and the technology could be extended to generate indicative element concentrations in polymetallic sulfide deposits in real-time [22,23]. Hyperspectral long-wave infrared images of Israeli soil were obtained and the emissivity spectrum for each sample was calculated [24]. Mineral-related emissivity characteristics were identified indicators and indices were created to determine quartz, clay minerals, and carbonate content in soils, which agreed with the mineralogical results obtained by chemical analysis. These studies demonstrate the high feasibility and accuracy of TIH technology in the quantitative analysis of various minerals with thermal infrared characteristics [21,25,26,27,28].

Although there have been numerous studies on using TIH to identify various mineral contents in soil, there is currently limited literature available on the identification of various salt contents [14,17]. Since TIH technology could capture feature information with hyperspectral to accurately detect salt mineral content [29], it is promising in detecting salt minerals that have a tendency to form dense mixtures. Based on this, this study utilized TIH technology to acquire and quantitatively analyze the emissivity data of potassium salt. The contributions of this study are as follows: (1) TIH technology was employed to obtain thermal infrared spectral characteristic information of potassium salt; (2) The thermal infrared spectrum characteristics of pure minerals in potassium salt were obtained and the potassium salt grade was detected by CARS-PLSR method.

## 2. Technical Route

**Figure 1:**
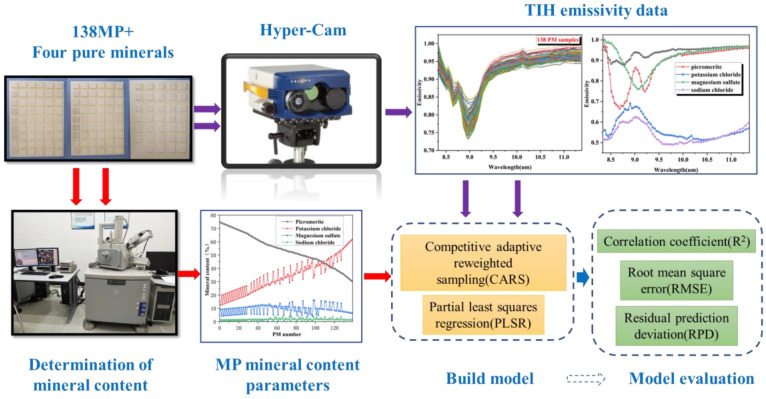
Schematic diagram of experimental and modeling process.

In this study, we aim to figure out the feasibility and accuracy of the TIH technique in the application of the mineral phases of potassium salts detection. The potash samples used in the study came from Xinjiang Lop Nur Potash Co., China’s largest potash sulfate producer. The main research process ware as follows: (1) Based on the actual MP samples, mixed potassium samples were designed and prepared by adding varying amounts of Pure minerals. A batch of potassium salt samples were obtained with sufficient and known mineral phase composition. (2) The samples were standardized by the pellet method, which stabilized the roughness of each sample and reduced the “noise” in the spectrum data. Subsequently, the spectral data of the potassium salt samples were collected using the Hyper-Cam hyperspectral imager in an open environment. (3) The TIH data for potassium salt was processed to obtain its emissivity data. Based on this emissivity data, prediction models were established for the mineral phase composition of potassium salt using the CARS and PLSR methods. (4) The prediction results of the model were evaluated and analyzed. The flowchart of the experimental and modeling process is shown in Figure 1.

## 3. MP Samples Preparation and TIH Data Acquisition

### 3.1. MP Sample Preparation

#### 3.1.1. MP Samples

Based on a random survey of 738 MP mineral phase data from the Lop Nur Potash production site in the first half of 2021, 138 MP samples of 5 g each were designed and configured. The 400 g MP sample from the production site of potassium salt (dried at 50 °C, mixed evenly by shifting cone method) was used as a master sample. The mineral phases and contents of the master sample were accurately analyzed with multiple detection methods. The 138 MP samples were then configured by adding one or more salts (contained in the MP) to the master sample.

#### 3.1.2. Mineral Facies Analysis

By comprehensive analysis of X-ray diffraction analysis (XRD) [30], MLA [31], Chemical titration, and ASS [32], the types and specific contents of various minerals in the MP master sample were defined. The specific parameters of the test method are as follows:

XRD: The D8 Advance X-ray diffractometer with Cu target from Bruker, Germany was used for XRD testing. The acceleration voltage is 40 kV and the anode current is 150 mA. Wide-angle diffraction patterns are collected in the range of 5–70° (2θ) at a scanning speed of 5°/min.

MLA: The system consists of a ZEISS Sigma model 300 high-resolution field emission scanning electron microscopy (FESEM), a Bruker XFlash 6|60 type X-ray spectrometer (EDS), and a set of AMICS software (including 4 subroutines, AMICSTool, Investigator, MineralSTDManager, and AMICSProcess). Experimental conditions: accelerating voltage 20 kV, High vacuum mode, a working distance of about 8.5 mm, backscattered electron detector (HDBSD), High Current mode. The sample was sprayed before testing to eliminate the charge on the surface of the non-conductive sample. The samples were polished using saturated MP mother liquor as a polish.

Chemical Titration:

Titration of K^+^: Sodium tetraphenylboron-quaternary amine salt volumetric method. Determination principle: Potassium ions react with sodium tetraphenolate boron to form potassium tetraphenolate boron deposits, and excess sodium tetraphenolate boron reacts with quaternary amine salt to form insoluble quaternary amine salt sodium tetraphenolate boron double salt.

Titration of Mg^2+^: EDTA complexometric titration. Determination principle: The cation in EDTA complexes with the calcium and magnesium cation in the test solution, so that the color of the solution changed from tartar red to azure blue, which was the endpoint.

Titration of ${\mathrm{SO}}_{4}^{2-}$: Moore method. Determination principle: Potassium chromate as indicator was titrated directly with silver nitrate standard solution in neutral weak alkaline solution.

Titration of Cl^-^: Barium chloride volumetric method. Determination principle: In an acidic solution (pH = 2~3), ${\mathrm{SO}}_{4}^{2-}$ and BaCl_2_ generated excessive barium salt precipitation and alizarin red~S indicator to generate a yellow complex, titrated with a known standard solution, adding ethanol to improve the sensitivity of the endpoint of the titration.

AAS:

Titration of Na^+^: According to the general rules of atomic absorption spectrometry, the sodium ions in the solution were analyzed by using the German continuous light source atomic absorption spectrometer at the pressure of 0.3 MPa.

#### 3.1.3. Mineral Composition of MP Samples

**Figure 2:**
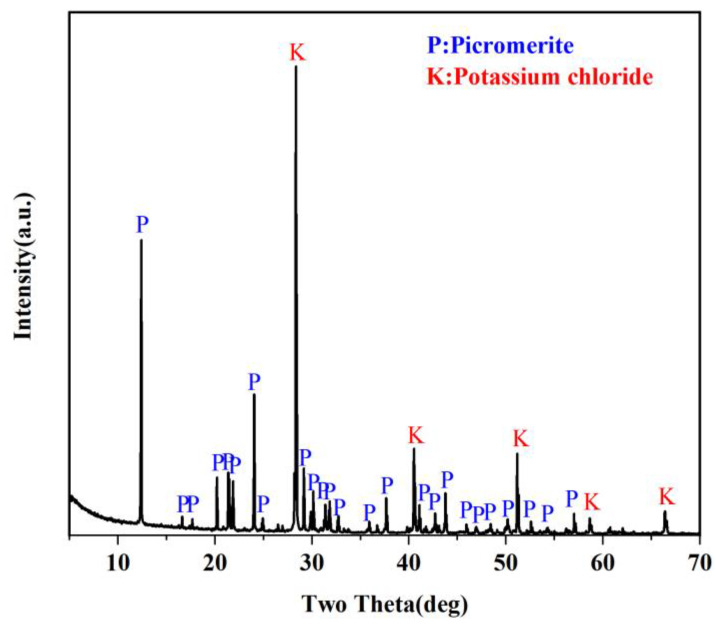
XRD data of MP sample.

**Figure 3:**
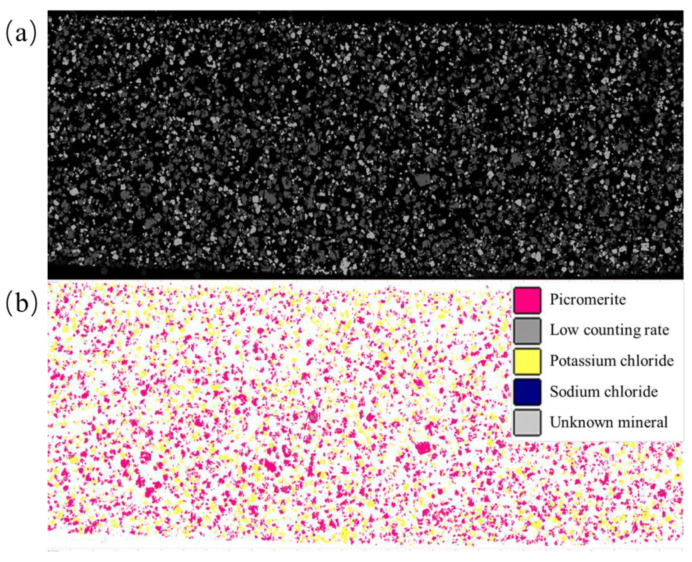
MLA test diagram of MP sample, (**a**) is the original image, and (**b**) is the processed image: the red area is picromerite, the dark gray area is low counting rate, the yellow area is potassium chloride, the blue area is sodium chloride, and light gray area is an unknown mineral.

XRD result in Figure 2 shows that the main components of MP are picromerite (KMg(SO_4_)_2_·6H_2_O) and potassium chloride (KCl). In the MLA test results shown in Figure 3, the element Mg:S:K in the red region has a mole ratio of approximately 1:2.03:1.85, indicating that picromerite is the primary mineral, followed by a small amount of magnesium sulfate (MgSO_4_·7H_2_O). The mole ratio of element K:Cl in the yellow region is about 1.01:1.00, which is expressed as KCl. The mole ratio of the element Na:Cl in blue is about 1.00:1.00, represented by sodium chloride (NaCl). The MLA detection result shows that the MP sample contains a large amount of picromerite and sodium chloride in addition to less potassium sulfate and potassium chloride.

However, during sample preparation for the MLA test, the MP sample was partially dissolved due to the requirement to wash and polish the sample simultaneously, despite the saturated MP mother liquor being used as a detergent. As shown in Table 1, 8.18% of the MLA test field is not uniform and could not be measured, which means, 5.37% of the MP sample could not be identified. Therefore, chemical volumetric methods were used to supplement the MLA results. The result of the chemical volumetric test in Figure 3 shows that the mass of potassium ion is 27.18%, a magnesium ion is 4.30%, sulfate ion is 33.67%, chloride ion is 13.57%, and sodium ion is 0.32%. Finally, the composition of the 400 g MP sample can be inferred as follows: picromerite, 65.70%, potassium chloride, 27.50%, magnesium sulfate, 5.97%, sodium chloride, 0.81%, combined with the ionic content data from the chemical titration in Table 2.

#### 3.1.4. Samples Library of MP

**Figure 4:**
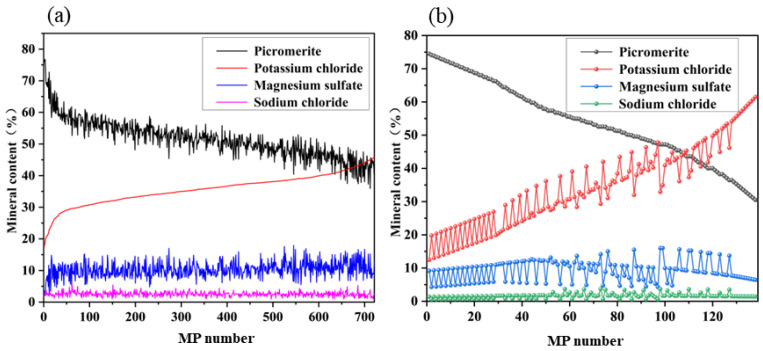
(**a**) Mineral composition of 738 MP samples from the production site. Mineral (**b**) composition of 138 MP samples designed and configured.

As shown in Figure 4a, among the 738 MP samples investigated, the content of picromerite ranged from 30.73% to 76.79%, with an average of 51.20% and a median of 51.19%. In 138 manually configured MP samples, the content parameters of picromerite ranged from 30.46% to 74.49%, with an average of 53.91% and a median of 53.43%. The content distribution of 138 MP samples was similar to that of 738 MP samples data. While Table 3 shows that potassium chloride, magnesium sulfate, and sodium chloride are also similar, which indicates 138 MP samples manually configured can completely meet the needs of field production.

### 3.2. TIH Data Acquisition

#### 3.2.1. Standardized Processing of MP Samples

The MP samples were then slightly ground and placed in 50 mL centrifuge tubes and mixed using a vortex oscillating mixer at 300 r/min for 6 min. Finally, the MP samples were successively put into a square mold with an inner diameter of 2 cm and pressed for 30 s under a pressure equivalent to 6 t to complete sample preparation.

#### 3.2.2. Hyperspectral Imaging System and Image Acquisition

In this study, a Hyper-Cam hyperspectral imager (Telops, Canada) was used for data acquisition, and its imaging mode was Fourier interference imaging. The spectral range of the instrument is 7.7–11.8 μm, the spectral resolution range is 0.25–150 cm^−1^, the Field of View (FOV) (°) is 40, and the imaging field is 320 × 256 array.

**Figure 5:**
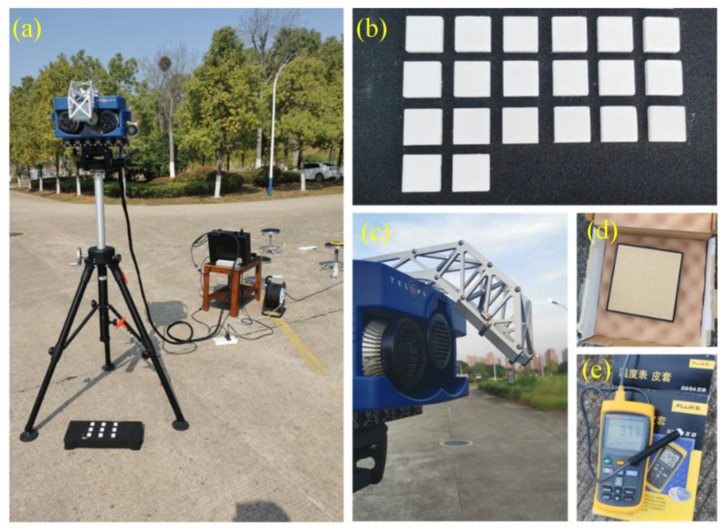
Conditions for collectingTIH data of potassium salt samples. (**a**) Sample shooting; (**b**) Potassium salt sample; (**c**) Hyper-Cam lens; (**d**) Gold-plate; (**e**) Thermometer.

As shown in Figure 5, the Hyper-Cam hyperspectral imaging system was used to collect TIH data from samples and a diffuse reflector (gold-plated plate) in a relatively open environment. The open environment can effectively avoid the interference of other objects in the surrounding environment and reduce the noise of acquiring data. At the same time, the sample was preheated with a heating table, and the heating temperature was set to 50 ℃. Fluke 54-IIB dual channel contact thermometer was used to record the temperature of the sample and gold foil before and after imaging, and the mean value was taken as the temperature of the sample and gold foil. Then, the absolute value of the difference between the measured temperature and the inverted temperature was used to evaluate the quality of the sample spectral data. It is generally believed that if the absolute value of the temperature difference is less than 1 K, the quality of collected sample data is acceptable and can be used for accurate quantitative analysis and study.

### 3.3. TIH Data Processing

#### 3.3.1. Emissivity Inversion

The method of obtaining sample emissivity in this study refers to the study [33]. Iterative Spectrally Smooth Temperature and Emissivity Separation (ISSTES) algorithm is widely used in remote sensing applications to accurately calculate the emissivity of solid objects from their spectral radiance measurements. The ISSTES takes advantage of the fact that the emissivity spectrum of solid objects is smoother than the characteristics of the atmosphere. ISSTES algorithm defines a group of emissivity curves indexed by surface temperature as follows:

(1) $$\varepsilon \left(\lambda \right)=\frac{L_{\mathrm{sensor}}\left(\lambda \right)-{\;L}_{u}\left(\lambda \right)-\;\tau \left(\lambda \right)E\left(\lambda \right)}{\tau \left(\lambda \right)B\left(\lambda ,\;T\right)-\;\tau \left(\lambda \right)E\left(\lambda \right)}$$

where L_sensor_(λ) is the total radiance reaching the sensor, λ is the central wavelength of each channel (μm), ε(λ) is the emissivity, and E(λ) is environmental radiance measured by the gold-plated plate. τ(λ) and Lu(λ) are atmospheric transmittance and upwelling radiation, respectively, which are simulated by MODTRAN^®^5.2. B(λ,T) is the Planck radiance at panel temperature T. The units for L_sensor_(λ), L_u_(λ), $B\left(\lambda ,\;T\right)$ and E(λ), are all W/(m²·sr·μm). ε(λ) and τ(λ) are dimensionless quantities.

As can be seen from Equation (1), given the surface temperature and known atmospheric parameters, the corresponding emissivity spectrum can be calculated. Then, if the smoothness criterion of the emissivity curve is determined, the smoothness of a series of emissivity curves at different temperatures near the real surface temperature can be calculated, and the temperature corresponding to the estimated emissivity spectrum is the smoothest result. The smoothness criterion used by ISSTES is designed as follows:

(2) $$S=\sum_{i=2}^{N-1}{\left(\varepsilon_{i}-\frac{\varepsilon_{i-1}+\varepsilon_{i}+\varepsilon_{i+1}}{3}\right)}^{2}$$

where S is the calculated smoothness criterion value, and N is the frequency band number of hyperspectral data.

In this study, the pretreatment of TIH data as well as the CARS and PLSR algorithms were calculated using MATLAB 2019a.

#### 3.3.2. Data Quality Evaluation by Temperature

**Figure 6:**
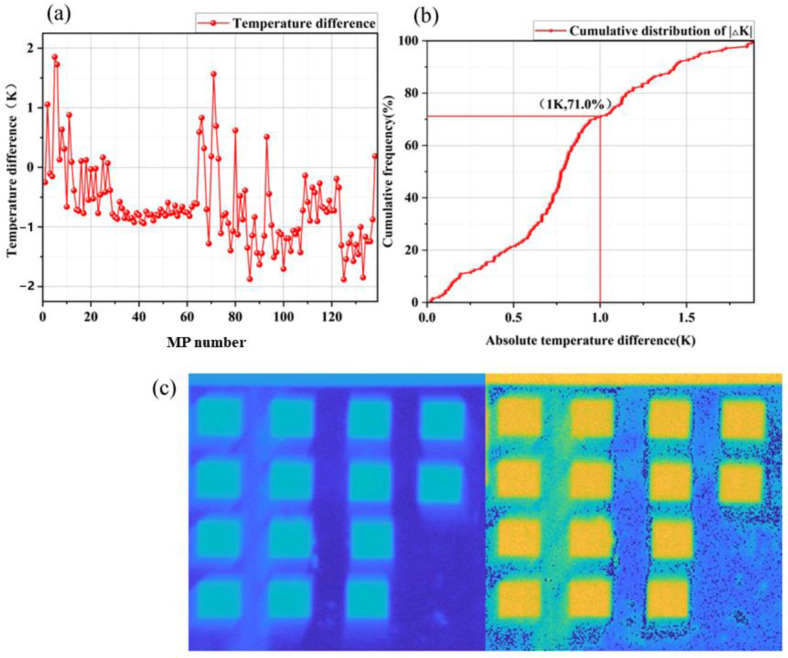
(**a**) Difference between the measured temperature and inverted temperature of 138 MP samples; (**b**) cumulative distribution of absolute temperature difference; (**c**) temperature profile of some MP samples.

In this study, the accuracy of the emissivity data was evaluated indirectly by comparing the measured temperature of the sample with the temperature obtained through inverted emissivity data using the algorithm. Since temperature and emissivity are coupled. When the emissivity error is about 0.015, the temperature error is about 1 k [34]. By comparing the measured and inverted temperatures, any discrepancies or errors in the emissivity data could be identified, thereby improving the accuracy of the spectral data and subsequent analysis. The difference between the measured and inverted temperatures was used as an indicator of the quality of the inverted data. This approach is crucial for the successful analysis and interpretation of the TIH data [35]. Figure 6a illustrates that the temperature difference of all samples is within ±2 K. In Figure 6b, 71.0% of the samples are in the range of absolute temperature difference ≤1 K, which indicates that the data collection and data processing process of MP samples is effective and the emissivity data obtained are of high quality. These emissivity results can be used for quantitative analysis and research of MP.

As can be seen from the temperature distribution of some MP samples in Figure 6c, the surface emissivity of MP samples is relatively uniform, indicating that MP samples are fully and evenly mixed during preparation. In addition, the mean emissivity of the 15 × 20 pixel at the center of the MP sample surface is taken as the emissivity of the corresponding sample to further reduce the effect of systematic errors in sample preparation.

## 4. Methods

### 4.1. Prediction Model

Firstly, 138 samples were tested for outliers and removed. To avoid some unreliable samples in the modeling calculation [36], the 138 samples were sorted and classified according to their picromerite content and then divided into 5 groups by regular interval sampling. Then, 4 groups of samples were randomly selected as the correction set, and the remaining group of samples was the prediction set. PLSR calculations were performed 5 times. Therefore, each sample had 4 calibration residual errors (CRE) and 1 predicted residual error (PRE). Then, PLSR [37] was used for analysis and calculation, and the CARS [38] method was introduced to choose feature wavelength. The PLSR model and the CARS-PLSR model were constructed separately. CARS were employed to screen out the sensitive bands corresponding to the minerals, while PLSR is utilized to establish a linear relationship between the mineral-sensitive bands and mineral content. The resulting model was then employed to estimate the content of salt ore.

The PLSR method, by projection, obtains orthogonal feature vectors for independent and dependent variables, respectively, and then establishes a unitary linear regression relation between the feature vectors. This approach not only resolves the issue of collinearity but also emphasizes the importance of independent variables in explaining and predicting dependent variables during the selection of feature vectors. By selecting several key variables, this method enables the model to achieve the minimum number of variables necessary for optimal performance. For the CARS method, the absolute value of the regression coefficient of the PLSR model is used as the index to evaluate the importance of each wavelength. In each sampling run, a fixed proportion of samples is randomly selected to establish the correction model. The number of samples selected in this study is 80% of the number of calibrations set. The wavelength for selection bands with larger absolute regression coefficients in the PLSR model is defined through adaptive reweighting sampling (ARS) technology. That is, CARS can often find the optimal combination of some key wavelengths that explain the chemical properties of interest.

### 4.2. Model Evaluation

To fully verify the performance of the model, the samples in the MP sample bank were divided into a calibration set and a testing set. The calculated parameters for the calibration set include the correlation coefficient ($R_{c}^{2}$) and root mean square error (RMSEC). The calculated parameters for the prediction set include the correlation coefficient ($R_{p}^{2}$), root mean square error (RMSEP), and relative prediction deviation (RPD). The higher $R_{c}^{2}$, $R_{p}^{2}$, and RPD, the lower RMSEC, and RMSEP, the better the predictive effect of the model. If $R_{c}^{2}$ is close to $R_{p}^{2}$, or RMSEC is close to RMSEP, the model is robust. In particular, an RPD value between 1.8 and 2.0 indicates a good model, between 2.0 and 2.5 indicates a very good model, and above 2.5 indicates excellent model performance [39].

(3) $$R^{2}=\frac{\sum_{i=1}^{n}{\left(\hat{y_{i}}-\bar{y}\right)}^{2}}{\sum_{i=1}^{n}{\left(y_{i}-\bar{y}\right)}^{2}}$$

(4) $$\mathrm{RMSE}=\sqrt{\frac{\sum_{i=1}^{n}{\left(\hat{y_{i}}-y_{i}\right)}^{2}}{n}}$$

(5) $$\mathrm{RPD}=\frac{\sqrt{\frac{\sum_{i=1}^{n}{\left(y_{i}-\bar{y}\right)}^{2}}{n-1}}}{\sqrt{\frac{\sum_{i=1}^{n}{\left(\hat{y_{i}}-y_{i}\right)}^{2}}{n}}}$$

In Formulas (3)–(5), $\hat{y_{i}}$ represents the predicted sample mineral content, $\hat{y_{i}}$ represents the mean value of the actual sample mineral content, $y_{i}$ represents the actual sample mineral content, and n represents the number of samples involved in the calculation.

## 5. Experiments and Results

### 5.1. Model Establishment for MP Prediction

#### 5.1.1. Outlier Detection

**Figure 7:**
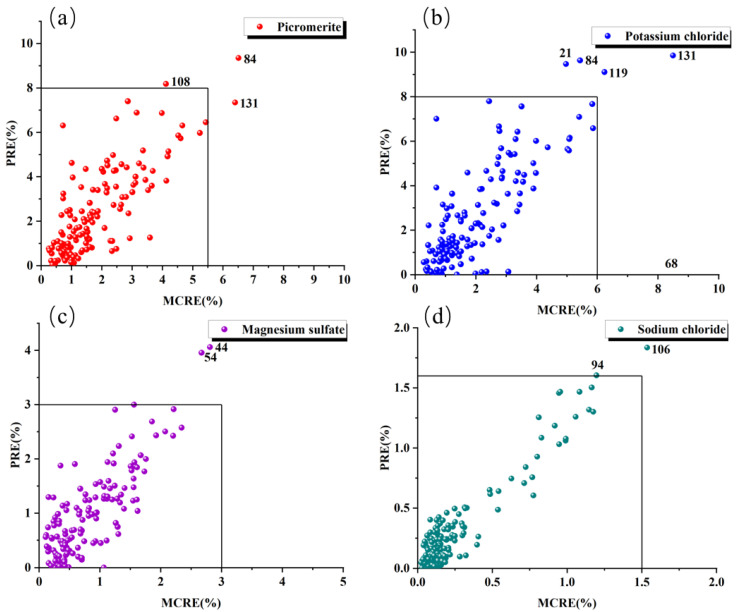
Anomalous sample detection of four minerals in MP samples. (**a**) picromerite content was used as the response variable; (**b**) potassium chloride content; (**c**) magnesium sulfate content; (**d**) sodium chloride content.

The outlier observation map of the MP sample was established using MCRE with abscissa and PRE with ordinate, as shown in Figure 7. The mean of corrected residual error (MPCE) and predicted residual error (PRE) were taken as the reference, and the larger samples were eliminated. Among them, the points with large MCRE and PRE values are removed. The samples with numbers 108, 84, and 131 are removed with picromerite; samples with numbers 21, 68, 84, 119, and 131 are removed with potassium chloride; samples with number 44 and 55 are removed with magnesium sulfate; samples with number 94 and 106 are removed with sodium chloride. That is, a total of 9 outliers are detected, and the remaining 129 points are used for subsequent model calculation.

#### 5.1.2. Calibration Set and Prediction Set

**Figure 8:**
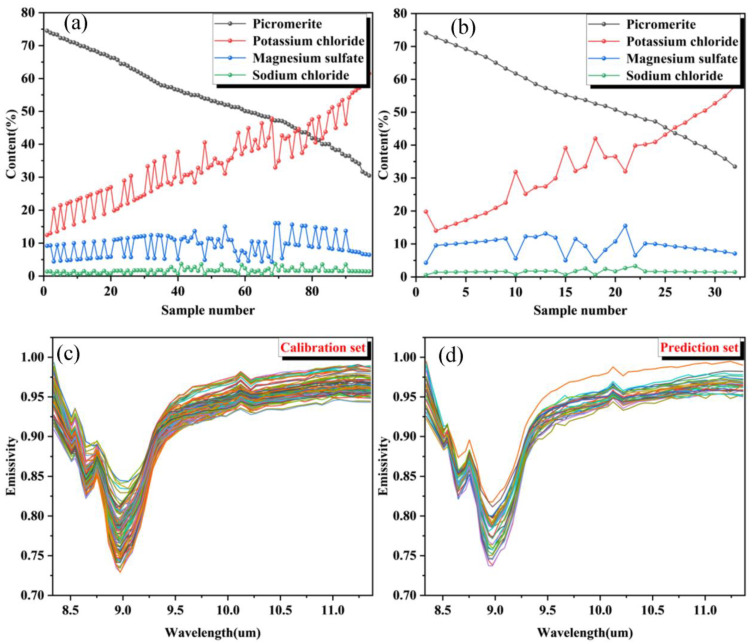
Mineral composition of the calibration set (**a**) and the prediction set (**b**); Emissivity data of the calibration set (**c**), and that of the prediction set (**d**).

As shown in Table 4 and Figure 8, a statistical analysis of the mineral composition of the four MP in the prediction and calibration sets is performed in terms of mean, standard deviation, minimum, median, and maximum. It illustrates that the range of sample parameters of the prediction set are all within the range of the calibration set. Moreover, from the two statistical terms mean and median, it can be found that the distribution of sample parameters of the prediction set and the calibration set is close, which means the results of the prediction set can fully illustrate the model performance of the calibration set.

### 5.2. MP Content Prediction

**Figure 9:**
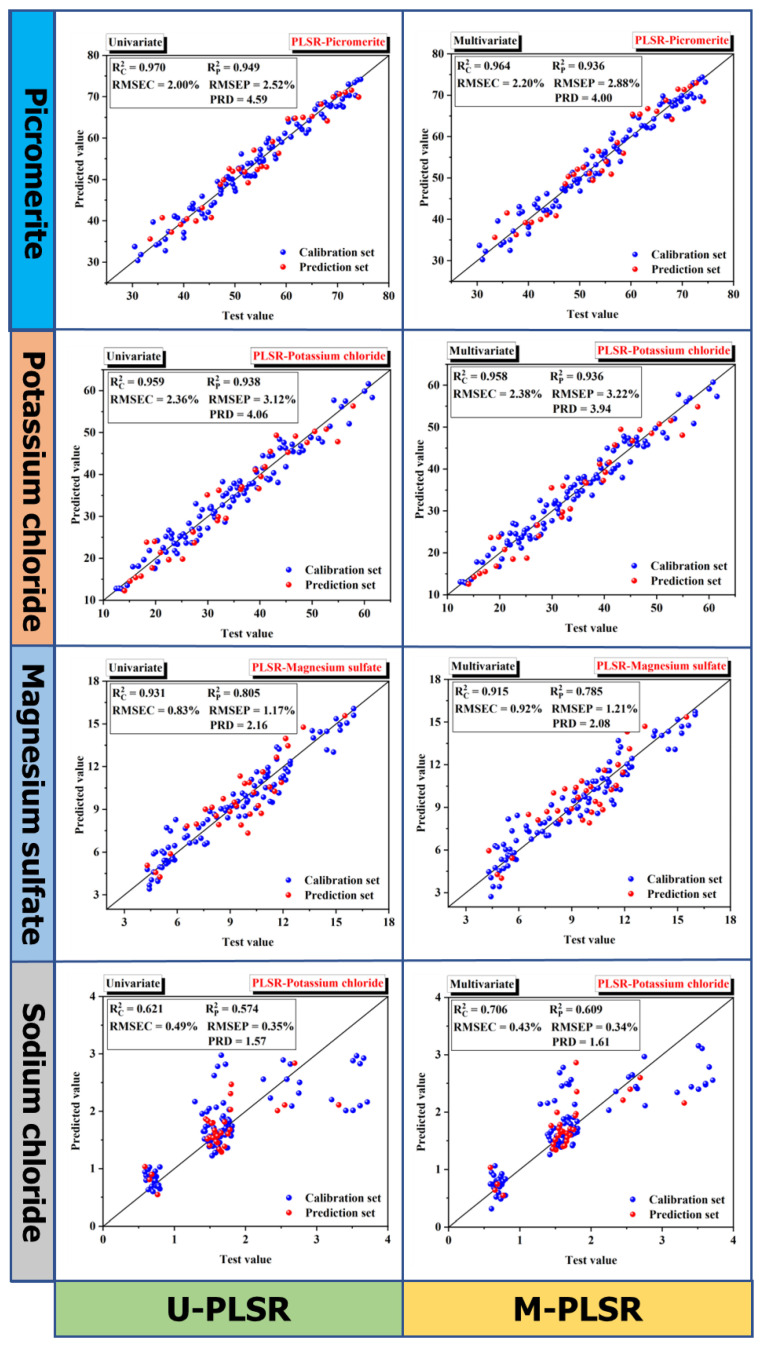
Prediction results of U-PLSR and M-PLSR models for MP samples.

In the calibration set, PLSR conducts internal validation of the model based on leave-one-out validation, and the calculation results are shown in Table 5 and Figure 9. PLSR is the calculation method of multivariate linear relationship analysis, and the relationship between the percentage content of the four minerals in the MP is not clear. Therefore, univariate PLSR (U-PLSR) and multivariate PLSR (U-PLSR) are used for calculation. From the comparison of RPD values of the prediction set, the prediction accuracy of U-PLSR is higher than that of M-PLSR. The RPD values of four minerals of U-PLSR are 4.59, 4.06, 2.16, and 1.57, respectively, and those of four minerals of M-PLSR are 4.00, 4.06, 2.08, and 1.57, respectively. In the process of PLSR calculation, the components extracted from the joint composition matrix Y of the four minerals cannot represent the various minerals themselves, which deduced that there should be no correlation between the percentage contents of the four minerals.

In the calibration set of U-PLSR, the $R_{c}^{2}$ of picromerite and potassium chloride is larger, while the $R_{c}^{2}$ of magnesium sulfate and sodium chloride is smaller, which may be related to the ratio of four minerals in the MP sample. It means that the content of picromerite and potassium chloride in the calibration set is greater than that of magnesium sulfate and sodium chloride. In MP samples, the signal strength may be more pronounced when the content of a particular mineral is larger, and the final prediction accuracy is higher. In the U-PLSR, the RMSEP values of the four minerals are 2.52%, 3.12%, 1.17%, and 0.35%, respectively, which increase by 0.52%, 0.76%, 0.34%, and 0.14% compared with the RMSEC values. The results indicate that the prediction set is accurate and the PLSR model is robust to the mineral content prediction of MP.

### 5.3. Sensitive Bands Selection of Pure Mineral

**Figure 10:**
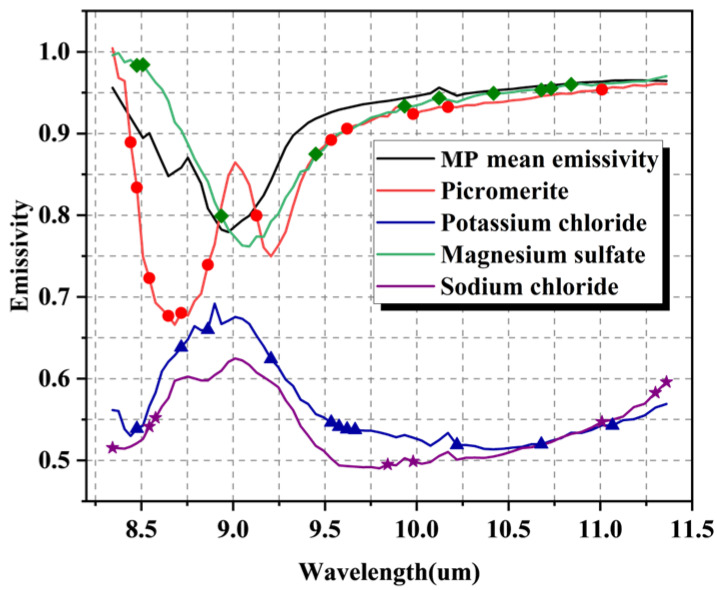
Sensitive wavelengths selected by the CARS method for four minerals in MP samples.

The CARS method is implemented through PLSR calculation of a single dependent variable, which can screen out the respective sensitive wavelengths of each mineral in MP samples. The CARS method could simplify the entire prediction method and enhance the feasibility of application in realistic scenarios of potassium salts. Table 6 shows that there are 12 sensitive wavelengths for picromerite, 11 sensitive wavelengths for potassium chloride, 11 sensitive wavelengths for magnesium sulfate, and 8 sensitive wavelengths for sodium chloride. Figure 10 maps and compares the sensitive wavelengths of each mineral, pure mineral, and MP mean emissivity spectrum data.

The sensitive wavelength distribution of picromerite is relatively uniform and distributed in the whole wavelength, among which there are four sensitive wavelengths concentrated in the trough of 8.5–9.0 μm. For the sensitive wavelengths of magnesium sulfate, the distribution of sensitive wavelengths is far away from the trough at 9.1 um, except for one near the trough at about 8.9 μm. The emissivity curves of potassium chloride and sodium chloride are similar in shape, with a broad peak between 8.5 and 9.5 μm, but the sensitive wavelengths do not overlap. The sensitive wavelength of potassium chloride is uniformly distributed over the entire thermal infrared band, while the sensitive wavelength of sodium chloride is mainly distributed on both sides of the thermal infrared band.

**Figure 11:**
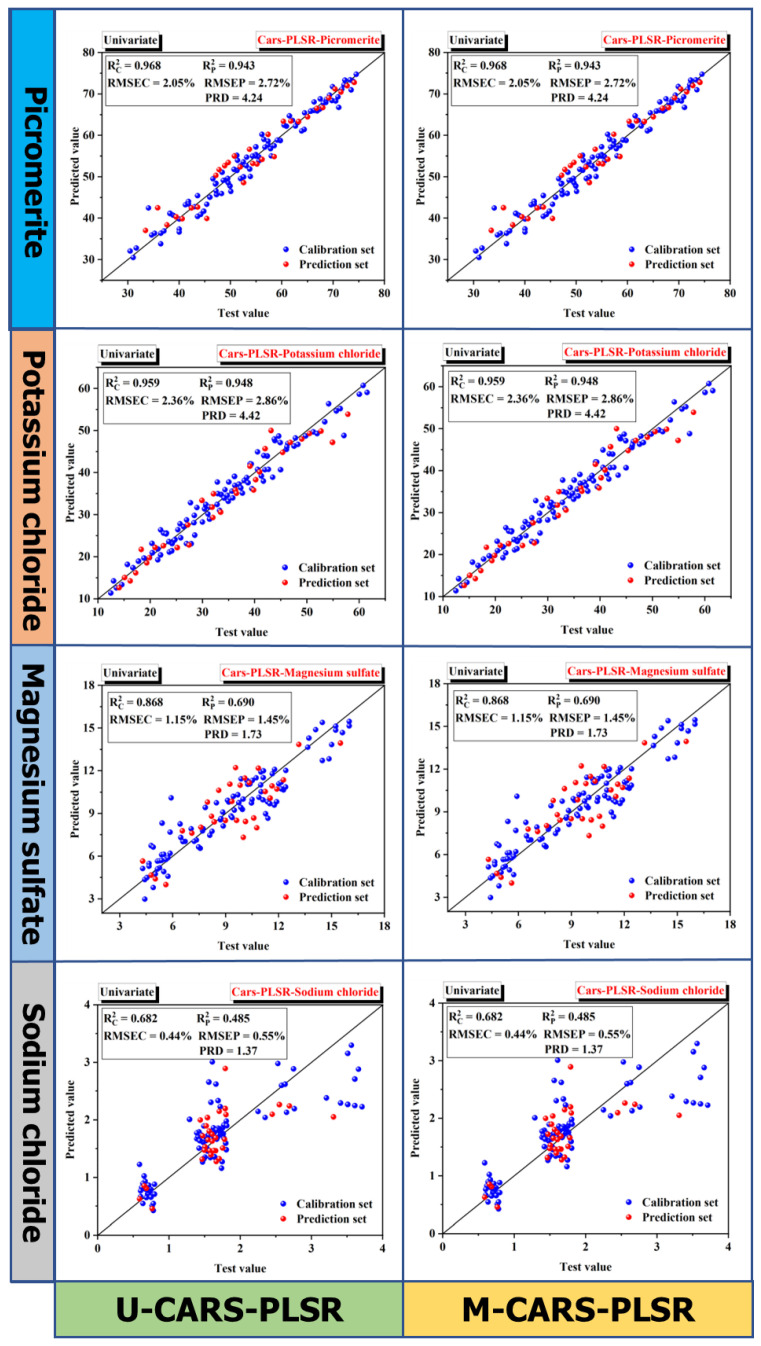
Prediction results of U- CARS -PLSR and M- CARS -PLSR models for MP samples.

Since the sensitive wavelengths selected by the CARS method for four minerals in MP overlaps, U-PLSR and M-CARS-PLSR methods are used to establish prediction models for U-CARS-PLSR and M-CARS-PLSR (the input wavelength is the union of the sensitive wavelengths of four minerals, 31 wavelengths), as shown in Figure 11 and Table 7. In the calibration set, the values of $R_{c}^{2}$ and RMSEC of the U-CARS-PLSR model are higher than those of the M-CARS-PLSR for picromerite and potassium chloride, while the results reverse for magnesium sulfate and sodium chloride. In the prediction set, the values of RMSEP and RPD for picromerite are almost the same (RMSEP: 2.72%, 2.78%, and RPD: 4.24, 4.15; RMSEP of sodium chloride: 0.40%, 0.40%, and RPD: 1.37, 1.36), but the results of potassium chloride and magnesium sulfate were significantly different (RMSEP of potassium chloride: 2.86%, 3.16%, and RPD: 4.42, 4.00; RMSEP of magnesium sulfate: 1.45%, 1.29%, and RPD: 1.73, 1.96).

**Figure 12:**
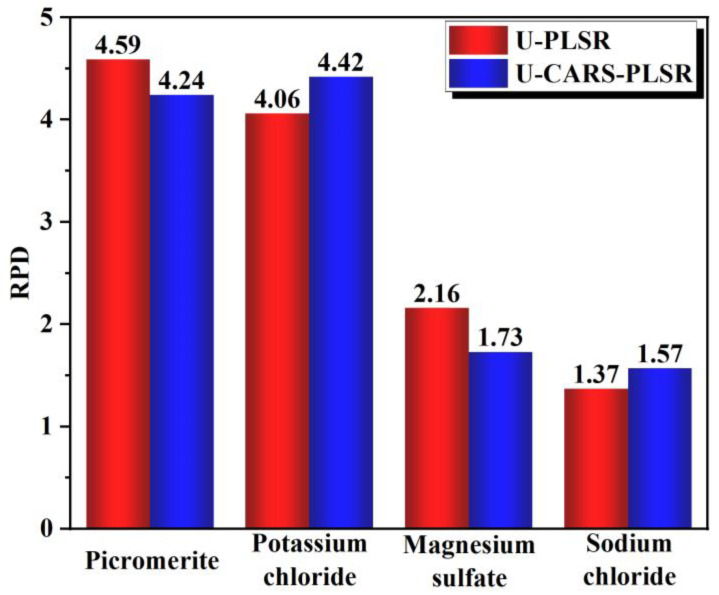
RPD values of four minerals in U-PLSR and U-PCARS-PLSR models.

Considering that the percentage of magnesium sulfate is much smaller than potassium chloride (standard deviation: potassium chloride, 12.66%; magnesium sulfate: 2.52%), it can be concluded that the performance of U-CARS-PLSR is better than that of M-CARS-PLSR. As Figure 12 shows, four minerals of RPD values of U-PLSR and U-CARS-PLSR are compared: picromerite 4.59, 4.24; potassium chloride, 4.06, 4.42; magnesium sulfate 2.16, 1.73; sodium chloride 1.37, 1.57. The results deduced that the prediction accuracy of U-CARS-PLSR has decreased to a certain extent except for potassium chloride. However, the CARS method reduced the number of selected calculated wavelengths (from 69 to 31 wavelengths), and the RPD values of major minerals in MP samples are both greater than 4.00, indicating that the prediction effect was still acceptable. In other words, CARS have a great role in reducing the cost of TIH technology and then being applied to the production and detection of potassium salt.

## 6. Conclusions

To solve the problem of rapid detection in the process of mineral production, this paper introduced the application of TIH technology in the detection of potassium salt samples as an example. The relationship between the potassium salt spectrum and mineral content was constructed, and the potassium salt content was successfully predicted under the PLSR method. The following conclusions are reached:

(1) TIH imaging revealed highly prominent emissivity characteristics of the MP samples in the thermal infrared band. Furthermore, the temperature discrepancy between the inverted and actual temperatures of the samples was relatively small, with 71% of the samples exhibiting a temperature difference of less than 1 K. This suggests that the emissivity accuracy of the samples obtained in this experimental process is high and can be approximated as the emissivity spectrum corresponding to potassium salt samples.
(2) The CARS-PLSR model, which is based on MP sample emissivity data training, is effective for MP sample prediction. In the U-PLSR model, the RPD values of the four minerals are 4.59, 4.06, 2.16, and 1.57, respectively, indicating that PLSR has a good prediction effect on MP. The calculation results of the U-CARS-PLSR model using the CARS method for sensitive wavelength selection show that the CARS method can effectively reduce the number of wavelengths, which is of great benefit to the practical application of TIH technology. With the CARS method, the number of selected wavelengths for the four minerals is reduced to 12, 11, 11, and 8, with RPD values of 4.24, 4.42, 1.73, and 1.37. The prediction accuracy of major minerals in MP is high (RPD > 4). The model has a good prediction effect on MP.

The study deduces that TIH technology has high accuracy for detecting potassium salts with limited mineral phases. It is reasonable to expect that TIH technology could have potential applications not only in detecting other minerals in production but also in other scenarios where mineral phases are limited, such as in the detection of allotropes.

TIH technology can accurately and rapidly detect mineral phases with limited mineral phases, compared with traditional detection methods. However, it is important to note that in this study, the TIH technology was used to collect data on dry potassium salt samples in a relatively open environment, which may be difficult to replicate under actual production conditions. Therefore, in subsequent studies, two approaches will be taken to address this issue: (1) Construct “shielding conditions” using low emissivity substances to simulate a more realistic environment; (2) Increase the sample size of potassium salt under aqueous conditions and quantitatively investigate the influence of water. These two approaches represent the focal points of follow-up research aimed at bringing the technology closer to practical application.

## Figures and Tables

**Table 1:** MLA analysis of the MP sample showed that picromerite accounted for 64.60%, potassium chloride 29.05%, sodium chloride 0.01%, low count rate 5.37%, and unknown minerals 0.96%.

Mineral Name	Weight (%)	Area (%)	Area (μm^2^)	Particle Number	Statistical Relative Error(%)
Picromerite	64.60	55.29	10,332,530.00	74,425.00	0.01
Potassium chloride	29.05	35.36	6,607,637.00	44,909.00	0.01
Low count rate	5.37	8.18	1,528,364.25	185,661.00	0.00
Unknown minerals	0.96	1.17	218,234.56	2525.00	0.04
Sodium chloride	0.01	0.01	1517.26	18.00	0.47

**Table 2:** Chemical titration data of MP sample.

Ion Species	K^+^	Mg^2+^	$SO_{4}^{2-}$	Cl^−^	Na^+^
Content(%)	27.18	4.30	33.67	13.57	0.32

**Table 3:** Mineral content parameter statistics of two groups of samples: group one, 738 survey MP data sets; group two, 138 manually configured MP sample data.

		Sum	Mean (%)	Standard Deviation (%)	Minimum (%)	Median (%)	Maximum (%)
Group one	Picromerite	738	51.20	6.14	30.73	51.19	76.79
	Potassium chloride		35.94	4.98	17.39	36.13	52.50
	Magnesium sulfate		10.31	2.43	2.25	10.11	17.93
	Sodium chloride		2.55	0.71	0.97	2.56	5.51
Group two	Picromerite	138	53.91	11.67	30.46	53.43	74.49
	Potassium chloride		34.59	12.05	12.46	33.88	61.52
	Magnesium sulfate		9.26	3.10	4.30	9.53	16.00
	Sodium chloride		1.65	0.75	0.59	1.59	3.73

**Table 4:** Mineral composition statistics from the calibration set (104 MP samples) and the prediction set (34 MP samples): mean, standard deviation, minimum, median, and maximum.

		Sum	Mean (%)	Standard Deviation (%)	Minimum (%)	Median (%)	Maximum (%)
Calibration set	Picromerite	97	54.05	11.67	30.46	53.76	74.49
	Potassium chloride		34.50	11.73	12.46	34.17	61.52
	Magnesium sulfate		9.24	3.23	4.30	9.43	16.00
	Sodium chloride		1.65	0.80	0.59	1.59	3.71
Prediction set	Picromerite	32	54.54	11.55	33.45	54.03	74.10
	Potassium chloride		33.68	12.66	14.05	32.80	57.84
	Magnesium sulfate		9.46	2.52	4.30	9.72	15.51
	Sodium chloride		1.64	0.55	0.59	1.61	3.31

**Table 5:** Results of calibration and prediction sets for U-PLSR and M-PLSR models.

		Calibration Set		Prediction Set		
		$R_{c}^{2}$	RMSEC	$R_{p}^{2}$	RMSEP	RPD
U-PLSR	Picromerite	0.970	2.00%	0.949	2.52%	4.59
	Potassium chloride	0.959	2.36%	0.938	3.12%	4.06
	Magnesium sulfate	0.931	0.83%	0.805	1.17%	2.16
	Sodium chloride	0.621	0.49%	0.574	0.35%	1.57
M-PLSR	Picromerite	0.964	2.20%	0.936	2.88%	4.00
	Potassium chloride	0.958	2.38%	0.936	3.22%	3.94
	Magnesium sulfate	0.915	0.92%	0.785	1.21%	2.08
	Sodium chloride	0.706	0.43%	0.609	0.34%	1.61

**Table 6:** CARS sensitive wavelengths screening results.

Mineral Species	Number	Sensitive Wavelengths (μm)
Picromerite	12	8.44, 8.48, 8.54, 8.65, 8.72, 8.86, 9.13, 9.53, 9.62, 9.98, 10.17, 11.01
Potassium chloride	11	8.48, 8.72, 8.86, 9.21, 9.53, 9.58, 9.62, 9.66, 10.22, 10.68, 11.07
Magnesium sulfate	11	8.48, 8.51, 8.94, 9.45, 9.62, 9.93, 10.12, 10.42, 10.68, 10.73, 10.84
Sodium chloride	8	8.34, 8.54, 8.58, 9.84, 9.98, 11.01, 11.30, 11.36

**Table 7:** Results of calibration and prediction sets for U-CARS-PLSR and M-CARS-PLSR models.

		Calibration Set		Prediction Set		
		$R_{c}^{2}$	RMSEC	$R_{p}^{2}$	RMSEP	RPD
U-CARS-PLSR	Picromerite	0.968	2.05%	0.943	2.72%	4.24
	Potassium chloride	0.959	2.36%	0.948	2.86%	4.42
	Magnesium sulfate	0.868	1.15%	0.690	1.45%	1.73
	Sodium chloride	0.682	0.44%	0.485	0.40%	1.37
M-CARS-PLSR	Picromerite	0.964	2.20%	0.940	2.78%	4.15
	Potassium chloride	0.955	2.47%	0.938	3.16%	4.00
	Magnesium sulfate	0.922	0.88%	0.770	1.29%	1.96
	Sodium chloride	0.715	0.42%	0.500	0.40%	1.36

## Data Availability

The data is unavailable due to privacy restrictions.
